# Bacterial siderophores efficiently provide iron to iron-starved tomato plants in hydroponics culture

**DOI:** 10.1007/s10482-013-9954-9

**Published:** 2013-06-29

**Authors:** W. Radzki, F. J. Gutierrez Mañero, E. Algar, J. A. Lucas García, A. García-Villaraco, B. Ramos Solano

**Affiliations:** 1 Department of Fruits, Vegetables and Mushrooms Technology, Faculty of Food Sciences and Biotechnology, Life Sciences University in Lublin, ul. Skromna 8, 20-704 Lublin, Poland; 2 Facultad de Farmacia, Universidad San Pablo CEU, Ctra Boadilla del Monte km 5.3, 28668 Madrid, Spain

**Keywords:** Iron, Siderophores, Plant growth-promoting rhizobacteria (PGPR), Biofertilizer, Tomato, Bacterial chelator

## Abstract

Iron is one of the essential elements for a proper plant development. Providing plants with an accessible form of iron is crucial when it is scant or unavailable in soils. Chemical chelates are the only current alternative and are highly stable in soils, therefore, posing a threat to drinking water. The aim of this investigation was to quantify siderophores produced by two bacterial strains and to determine if these bacterial siderophores would palliate chlorotic symptoms of iron-starved tomato plants. For this purpose, siderophore production in MM9 medium by two selected bacterial strains was quantified, and the best was used for biological assay. Bacterial culture media free of bacteria (S) and with bacterial cells (BS), both supplemented with Fe were delivered to 12-week-old plants grown under iron starvation in hydroponic conditions; controls with full Hoagland solution, iron-free Hoagland solution and water were also conducted. Treatments were applied twice along the experiment, with a week in between. At harvest, plant yield, chlorophyll content and nutritional status in leaves were measured. Both the bacterial siderophore treatments significantly increased plant yield, chlorophyll and iron content over the positive controls with full Hoagland solution, indicating that siderophores are effective in providing Fe to the plant, either with or without the presence of bacteria. In summary, siderophores from strain *Chryseobacterium* C138 are effective in supplying Fe to iron-starved tomato plants by the roots, either with or without the presence of bacteria. Based on the amount of siderophores produced, an effective and economically feasible organic Fe chelator could be developed.

## Introduction

Iron is a crucial element for proper plant development. Since it is a cofactor of many metabolic pathways its deficiency may lead to disruption of many processes including respiration or photosynthesis and be the reason for a chlorosis in the aftermath (Guerinot 2010; Zuo and Zhang 2011). Iron is the fourth most abundant element in the earth’s crust and in most types of soil occurs in excess. This element can exist in aqueous solution in two states: Fe^2+^ and Fe^3+^; however, Fe^3+^ forms are not readily utilizable by plants and microbes because they often form insoluble oxides or hydroxides which limit bioavailability (Desai and Archana 2011; Zuo and Zhang 2011). It is estimated that approximately one-third of earth’s soil can be considered iron deficient (Yi et al. 1994).

Plants use two strategies to acquire iron. Strategy I involves acidification of the rhizosphere followed by reduction of Fe^3+^ ions by membrane-bound Fe(III)-chelate reductase and subsequent uptake of Fe^2+^ into root cells. Strategy II refers to the plants which secrete low molecular weight phytosiderophores in order to solubilise and bind iron which is then transported into root cells via membrane proteins (Altomare and Tringovska 2011; Guerinot 2010). However, these strategies are often not efficient enough to meet the needs of the plants growing especially in calcareous and alkaline soils. Therefore, providing plants with accessible forms of iron is necessary when it is scant or unavailable in soils (Zuo and Zhang 2011).

The amount of iron absorbed by plants is also important for human health. According to World Health Organization, approximately two billion people around the world are anaemic, mainly due to iron deficiency. This problem could be approached by consumption of iron-rich fruits and vegetables (www.who.int/nutrition/topics/ida/en/index.html). Tomato ranks third by weight in global production of fruits and vegetables and tomato fruits or products are widely consumed all over the world reaching 40 kg per capita on average in the United States (Tan et al. 2010). Tomato fruits possess high nutritional value as a source of many essential minerals and vitamins like vitamin C, vitamin E, folate and potassium. It also contains substantial amounts of carotenoids, especially lycopene and β-carotene which have high antioxidant potential and contribute to anticancer activity (Adalid et al. 2010; Campbell and Erdman 2005; Tan et al. 2010).

Generally, there are two main strategies to deliver iron to the plants—soil and crop management. Crop management practices involve foliar and root delivery of iron in inorganic form (FeSO_4_) or as synthetic or non-synthetic Fe-chelates (Fernández et al. 2005; Godsey et al. 2003). It is noteworthy that applying Fe-chelates to leaves usually gives more satisfactory results compared to an inorganic form of iron (Vempati and Loeppert 1988). Soil management may involve fertilizing with inorganic salts (as NO_3_^−^ or NH_4_^+^) in order to change pH, thus improving iron solubility and increasing its uptake by the plants (Zuo and Zhang 2011). A second way of soil management is based on delivering iron to soil as chelates or FeSO_4_. However, using synthetic chelates like Fe-EDTA or Fe-EDDHA is cheaper and more effective (Shenker and Chen 2005) but their overuse may have negative environmental impacts (Adesemoye et al. 2009).

Another method of fertilizing soil, which is currently gaining importance since it is considered environmentally friendly, is the application of inoculants containing one or more beneficial microorganisms known as plant growth-promoting rhizobacteria (PGPR). Among the benefits for plant fitness attributed to PGPR is the ability to release siderophores, compounds below 2 kDa capable of chelating Fe with high affinity and in a reversible manner (Budzikiewicz 2010; Neilands 1995). The functional groups responsible for the binding are most frequently hydroxymates and catechols (Raymond et al. 1984). Many studies demonstrated that microbial siderophores are used by plants (Crowley et al. 1992; Fernández et al. 2005; Johnson et al. 2002). Furthermore, many PGPR strains exert additional beneficial effects on the plants apart from improving iron nutrition, since they are able to solubilise phosphates and other micronutrients in soil (Ramos Solano et al. 2008). Moreover, some PGPR are able to release molecules identical to plant growth regulators, that are absorbed by the plant and, therefore, may cause increases in root surface, and subsequent enhanced nutrient uptake (Adesemoye et al. 2009; Ramos Solano et al. 2008; Ramos-Solano et al. 2010). However, the use of biofertilizers is not consistent yet in any field condition since bacterial genes are highly inducible and a number of factors may affect bacterial performance (Rainey 1999). Therefore, in order to overcome potential bacterial failure, the use of bacterial metabolites appears as an encouraging alternative.

The aims of this work were, therefore, (i) first, to evaluate bacterial siderophore production of two PGPR strains in vitro and (ii) to assess the effect of bacterial siderophores supplemented with iron alone (metabolite) and in combination with the PGPR (metabolite + cells) to overcome iron deficiency on iron-starved tomato plants (*Lycopersicon esculentum* var. Marglobe).

## Materials and methods

### Bacterial strains

Two siderophore-producing bacterial strains were studied; *Chryseobacterium* spp. C138 isolated from the rhizosphere of *Oryza sativa* (unpublished) and *Pseudomonas fluorescens* N21.4 from the rhizosphere of *Nicotiana glauca* (Ramos-Solano et al. 2010). Strains were kept on Triptone Soy Broth (TSB) amended with 15 % glycerol at −20 °C. Prior to experiment, bacteria were streaked on plate count agar (PCA) plates and grown for 24 h at 28 °C.

### Siderophore production media, quantitative determination and iron-binding capacity

Bacterial strains were grown in modified M9 medium (MM9) without added iron (Alexander and Zuberer 1991). The composition of this media was KH_2_PO_4_ 0.3 g, NaCl 0.5 g, NH_4_Cl 1 g, MgSO_4_·7H_2_O 493 mg, CaCl_2_ 11 mg, MnSO_4_·H_2_O 1.17 mg, H_3_BO_3_ 1.4 mg, CuSO_4_·5H_2_O 0.04 mg, ZnSO_4_·7H_2_O 1.2 mg, Na_2_MoO_4_·2H_2_O 1 mg, casamino acids 3 g, PIPES 30.24 g, FeCl_3_·6H_2_O 10 μM, EDTA 3.27 mg and glycerol 5 g. Siderophores were measured using modified chromeazurol S (CAS) assay (Alexander and Zuberer 1991). Deferoxamine mesylate (DFOM) (Sigma-Aldrich) was used as a standard to construct the calibration curve. Serial dilutions of the standard were mixed with CAS assay solution (1/1 v/v) and incubated for 4 h at 25 °C, prior to the spectrophotometric measurement at 630 nm done by double-beam UV–Vis spectrophotometer (Thermo, Biomate). Results are expressed as μM equivalents of DFOM. To calculate the iron-binding capacity of bacterial siderophores, 1 mmol of DFOM is considered to be able to bind 56 mg Fe^3+^.

### Quantification of bacterial siderophores

For each strain, a pre-inoculum was prepared from a 24-h grown PCA plate suspended in 10 mM magnesium sulphate buffer pH 6.8, with an optical density of 0.7 at 600 nm. One mL of the suspension was transferred to 250 mL Erlenmeyer flasks containing 100 mL of the MM9 media described above. Flasks were shaken at 175 rpm for 72 h at 28 °C.

Bacterial growth parameters (optical density at 600 nm, pH) and siderophore content were checked 3 times per day, at 3-h intervals. Prior to each determination, culture media were centrifuged at 4,000 rpm for 10 min at 4 °C and filtered through 0.2 μm (Millipore) to remove bacterial cells. Samples were stored at −20 °C until analysis. UV–Vis spectra of the culture supernatants, withdrawn after 44 h, were recorded on double-beam spectrophotometer (Biomate, Thermo) in the range of 220–500 nm. Siderophore content in bacterial supernatants was measured as described in "Siderophore production media, quantitative determination and iron-binding capacity" section, and absorbance values at 630 nm were interpolated on the calibration curve.

### Production of iron-bound bacterial siderophores

For the biological assay, 1 L of MM9 media was inoculated with 1 mL of bacterial suspension (10^8^ cfu mL^−1^). Flasks were shaken for 42 h at 28 °C at 175 rpm to allow siderophore production. Then, 500 mL was devoted to obtain siderophores alone (S) and 500 mL for siderophores and bacterial cells (SB). For siderophores alone (S), media were centrifuged at 4,000 rpm at 4 °C and filtered to remove bacterial cells. The second part (SB) contained siderophores and bacterial cells (10^9^ cfu mL^−1^). Siderophore content was checked as described in "Siderophore production media, quantitative determination and iron-binding capacity" section. Then, both parts were incubated with 19 μM of FeCl_3_·6H_2_O for 90 min at room temperature, to provide an equivalent amount of iron present in full Hoagland solution (1.064 μg/mL).

### Experimental design

Tomato seeds of *Lycopersicon esculentum* var. Marglobe were used. Plants were grown in a greenhouse under controlled conditions (15/9 h light/dark, 25/20 °C, R.H 20–70 %). Seeds were sown in 100 mL pots filled with sterile sand; pots were covered with plastic to ensure proper humidity for germination. When seedlings emerged, plastic wrap was removed and seedlings were watered with 10 mL per day; to ensure plant growth and chlorosis development, water was replaced by iron-free Hoagland solution every 3 days throughout the experiment. After 14 days, seedlings were divided into 5 groups (*n* = 9). Thirty-five days after the emergence, when the upper leaves turned yellow, treatments were delivered as follows: full Hoagland solution (positive control), iron-free Hoagland solution (negative control), siderophores (S) + Fe and siderophores and bacteria (SB) + Fe; the fifth group was kept only with water as a second negative control. Treatments (10 mL) were delivered twice, with a week in between. Seven days after treatment delivery, plants were harvested and dried for further nutrient determination. The following determinations were done: biometrical analyses (shoot length and stem diameter, shoot dry weight) and chlorophyll content determination.

For nutritional analyses, plants from each treatment were pooled, and analyses were carried out on the mixture of expanded upper leaves of each plant. Total N was analyzed by Kjeldhal digestion. Concentrations of P, S, Ca, Mg, K, Fe, Mn and Al were determined by HNO_3_/pressure digestion as described by Chander et al. (2008), followed by ICP-AES analysis (Spectro Analytical Instruments, Kleve, Germany). Nutritional analyses were carried out by Albion Laboratories.

### Chlorophyll determination

Chlorophyll content was determined on the last fully expanded leaf of plants from each treatment. Determination was done according to Lichtenthaler and Buschmann (2001). Chlorophyll was extracted with methanol from a stripe cut from a fully expanded upper leaf of each plant homogenized with a blender and the absorbance was measured at 665 and 652 nm.

### Statistical analysis

The results were statistically tested using unidirectional analysis of variance ANOVA with a level of significance set at *α* = 0.05. When differences were significant a Tukey test (*p* < 0.05) was performed with the computer programme Statgraphics plus 5.1, for WindowsTM.

## Results

### Bacterial growth and siderophore production

**Fig. 1:**
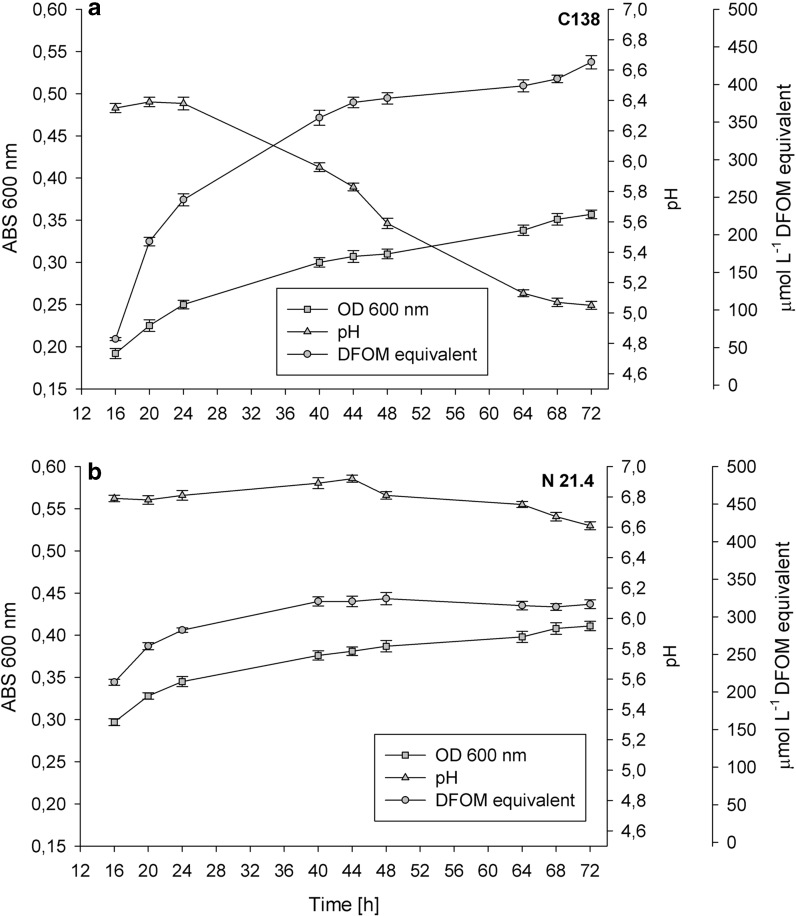
Growth parameters (bacterial density and pH) and siderophore production (μmol L^−1^ DFOM equivalent) by strains C138 (**a**) and N21.4 (**b**). Data are given as mean ± SD, *n* = 3

Siderophore production increased as bacterial biomass increased after 16 h of culture; the greatest accumulation of siderophores occurred at the stationary phase, after 40 h by strain N21.4, and after 72 h by strain C138 (Fig. 1). While strain C138 accumulated up to 425 μmol equivalents of DFOM in 72 h; strain N21.4 achieved lower production, reaching 300 μmol equivalents of DFOM in 40 h. During the incubation period, pH decreased with C138 by 1.4 U to values of 5, while it remained constant around 6.6 in N21.4 cultures. Siderophore production by C138 was more abundant and, therefore, it was selected for further experiments.

**Fig. 2:**
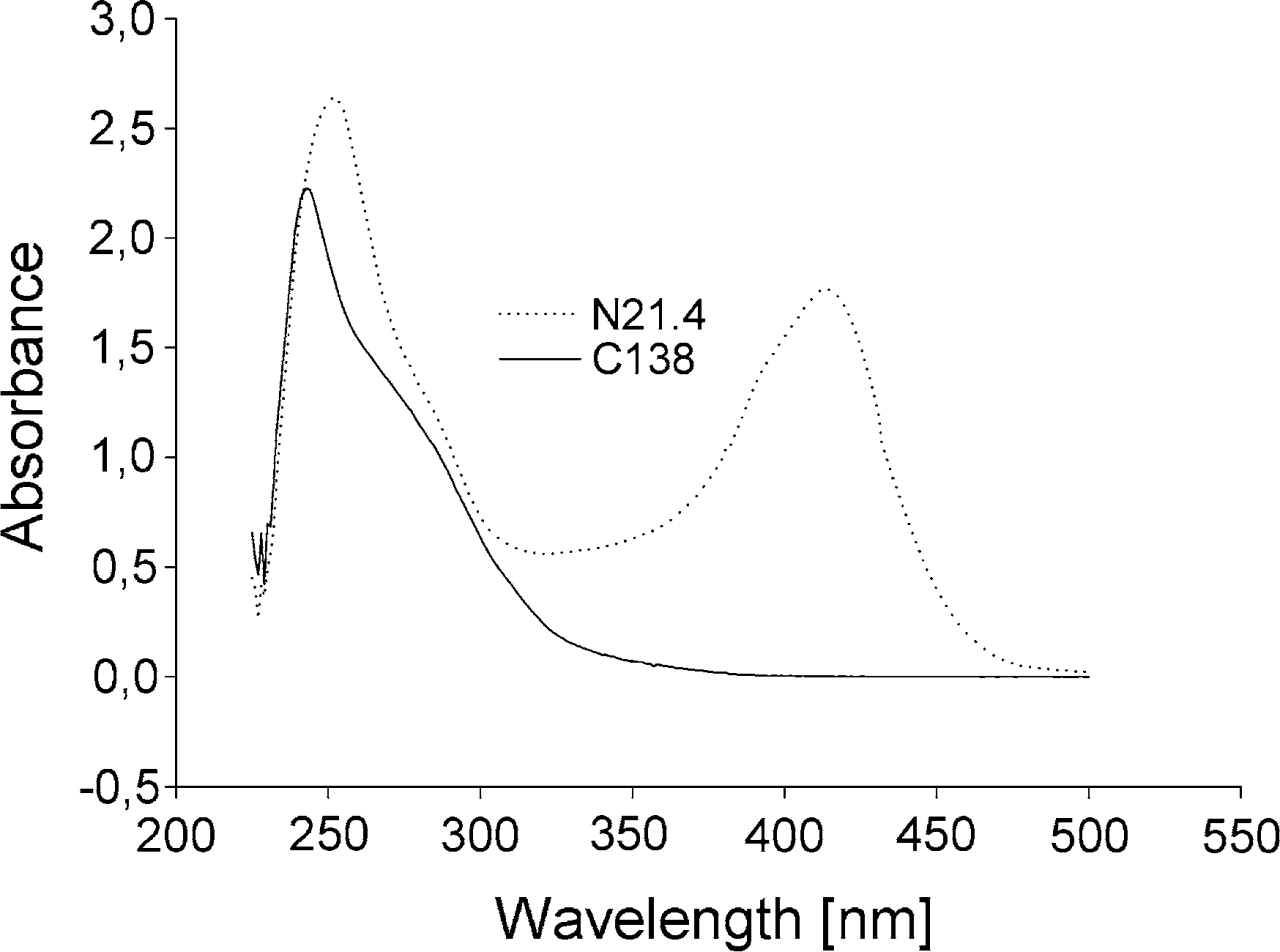
UV–Vis spectra of C138 and N21.4 siderophore-rich culture filtrates

UV–Vis spectra of bacterial culture filtrates revealed that the nature of the siderophore produced by each strain was different, while C138 filtrate does not absorb light in visible region while N21.4 does (Fig. 2).

### Biometrical analysis

**Fig. 3:**
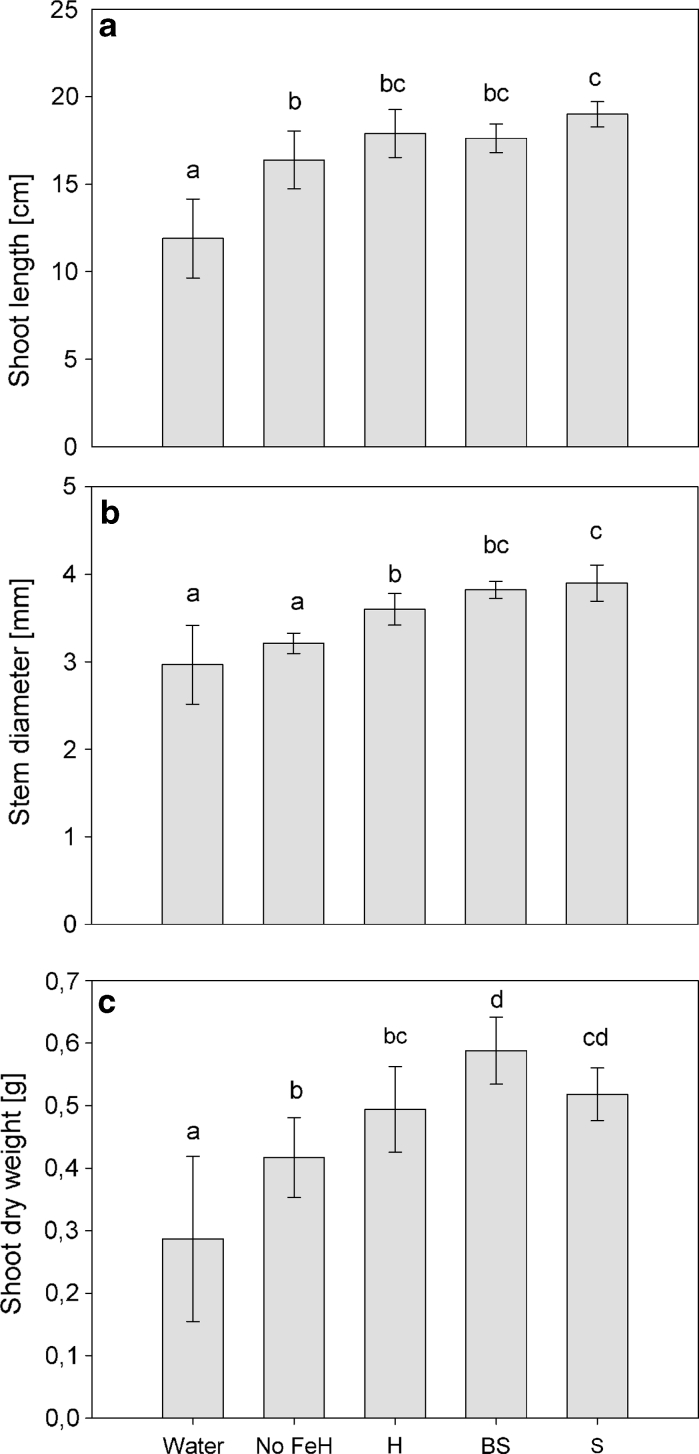
Biometrical parameters **a** shoot length (cm), **b** stem diameter (mm) and **c** shoot dry weight (g) of 70-day-old iron-starved tomato plants treated with C138 siderophores (S) and the siderophores + bacteria (BS), both supplemented with iron, and control plants (Water), full Hoagland solution (H), iron-free Hoagland solution (No FeH), 7 days after the second dose. Data represent mean values of nine replicates ± SE. *Different letters* indicate the existence of significant differences according to Tukey test (*α* = 0.05)

Data obtained from biometrical analysis of tomato plants appear in Fig. 3. Bacterial siderophores with (BS) or without bacteria (S), both supplemented with Fe affected biometrical features of plants. Values of stem diameter and shoot dry weight were significantly higher in treated plants than in the negative controls (iron-free Hoagland solution and water). Stem diameter (Fig. 3b) reached almost the same values (3.8 mm) with both bacterial treatments (S and BS) and only the S treatment was significantly higher than that obtained on plants watered with full Hoagland solution; no significant differences appeared between both negative control treatments (water-treated plants and iron-free Hoagland). Shoot length and weight were affected differentially by the siderophore (S) or the bacteria (BS). Shoot length (Fig. 3a) averaged 17 cm in iron-supplied plants (S, BS and H) being highest in plants treated with the siderophore (S). Conversely, shoot dry weight was highest on bacterial (BS)-treated plants (0.6 g) and significantly different from full Hoagland solution-treated plants (0.5 g). Interestingly, delivering living bacteria (BS) to the plants resulted in a significant increase in shoot dry weight, comparing to full Hoagland solution-treated plants (H).

### Chlorophyll content

**Fig. 4:**
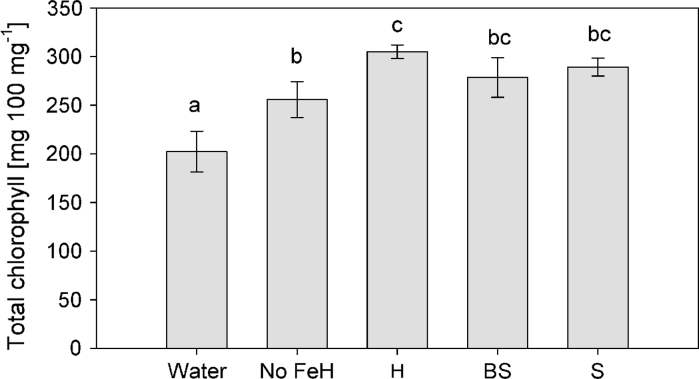
Total chlorophyll content in 70-day-old iron-starved tomato plants treated with C138 siderophores (BS) and the siderophores (S), both supplemented with iron, and control plants (Water), full Hoagland solution (H), iron-free Hoagland solution (no FeH), 7 days after the second dose. Data represent mean values of nine replicates ± SE. *Different letters* indicate the existence of significant differences according to Tukey test (*α* = 0.05)

Chlorophyll content analysis showed non-significant differences in total chlorophylls between siderophore treatments (S or BS) and the Hoagland-treated plants (Fig. 4), being the highest values in full Hoagland solution (H)-treated plants.

**Fig. 5:**
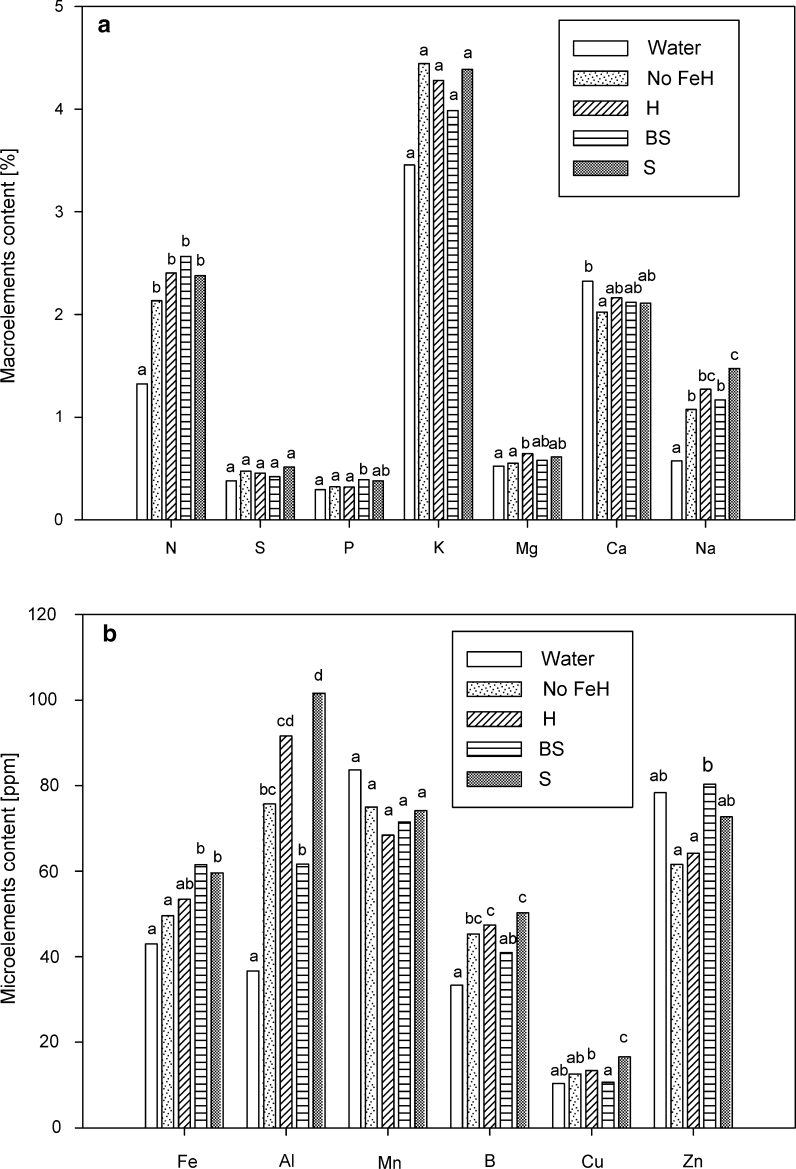
Nutritional status of 70-day-old iron-starved tomato plants treated with siderophores and bacteria (BS) and siderophores (S), both supplemented with iron, and control plants (Water), full Hoagland solution (Hoagland), iron-free Hoagland solution (no FeH) 7 days after the second dose. **a** macroelements (%) and **b** microelements (ppm). *Different letters* indicate the existence of significant differences according to Tukey test (*α* = 0.05)

The nutritional status of plants is shown in Fig. 5. BS- and S-treated plants contained the highest levels of iron (62 and 60 ppm, respectively), compared to full Hoagland solution-treated plants (53 ppm). The amounts of copper, boron, aluminium and sodium were highest in S-treated plants. Interestingly, aluminium uptake was moderate under bacterial influence and peaked on S-treated plants.

## Discussion

Iron is an essential element for plants, and, therefore, it is absolutely necessary to close plant cycle. Hence, relevance in agriculture is beyond any doubt, especially since its solubility is conditioned by pH. Iron deficiency has an important economic impact on the fruit sector because it can reduce fruit yield and quality (Álvarez-Fernández et al. 2006), and also because Fe fertilization is expensive (Rombolá and Tagliavini 2006). Up to the date, only synthetic chelates are available for agriculture and among these, EDTA and EDDHA are the most widely distributed (Orera et al. 2009). Both are poorly biodegradable and pose a threat to environment (Fernández et al. 2005), the main risk being the accumulation in ground water (Kaparullina et al. 2011). Although the high concentrations of EDTA itself are not toxic for humans, the main risk remains in its ability to bind heavy and toxic metals and rend them water soluble, therefore, contaminating drinking water (Kaparullina et al. 2011). Furthermore, EDTA is not easily removed during wastewater treatment (Kari and Giger 1995). In view of this, it is vital to find alternative ways to deliver iron to the plants which are sensitive to iron deficiency. Furthermore, organic farming and ecological agriculture call for a natural product able to cover up the needs in these handling procedures.

Production of siderophores is a frequent bacterial trait due to the physiological role of metals in cells, and their production is subject to metal availability/demand (Schalk et al. 2011), and, therefore, basal production is low. In this experiment, the production of organic chelates by two PGPR strains was evaluated in an iron-free medium, seeking the induction of siderophore production. The two strains were selected for their excellent results obtained on CAS agar medium plates (Ramos-Solano et al. 2010; unpublished). After evaluating the ability of each strain to release siderophores into culture media, strain C138 was able to produce high amounts of siderophores within 72 h, and, therefore, it was selected for the biological assay on tomato plants. The yellow-orange halos on CAS agar medium produced by C138 and the quick change of colour of CAS reagent suggest that it is of the catechol type (Schwyn and Neilands 1987). However, its chemical structure has not been determined yet. According to UV–Vis spectra of bacterial culture filtrates (Fig. 2), C138 filtrate does not absorb light in visible region compared to N21.4, which point out a difference in the chemical nature of the siderophore produced by these strains consistent with the reported structural variability for these group of compounds (Hider and Kong 2010).

Bioassay on iron-starved tomato plants revealed that iron-chelated bacterial siderophores were suitable for plants. Plants treated with bacterial chelates (S and BS) showed better growth parameters than plants deprived of any Fe source, reaching similar values than plants supplied with full Hoagland solution (Fig. 3), confirming the efficacy of bacterial siderophores. Interestingly, delivering siderophores and bacteria to plants (BS) resulted in a significant increase in shoot dry weight, compared to full Hoagland solution (H), and higher than plants treated only with siderophores (S), indicating that the bacteria are conferring an additional benefit to the plant other than providing Fe-chelates. Although in vitro test for auxin production or ACC degradation was recorded negative for C138 (unpublished), production of plant growth regulators is within the putative mechanisms of plant growth promotion. Despite the negative record for auxin production by C138, it is a fact that the effective amount of a plant growth regulator is conditioned by the plant species, physiological status and concentration of other plant growth regulators (Peleg and Blumwald 2011). Therefore, this strain may be altering a plant’s hormonal balance, which may affect root growth patterns and, therefore, improve nutrient absorption by increasing absorption area (Ramos-Solano et al. 2010).

Plant dry matter was well correlated with chlorophyll concentration in the upper leaves (Fig. 4), confirming that the chlorotic symptoms evidenced prior to treatment delivery due to iron deficiency were overcome with the bacterial siderophores. Interestingly, the presence of bacteria + siderophores (BS) in plant roots caused a significant increase of shoot dry weight compared to plants treated only with siderophores (S) supporting the proposed hypothesis of the bacteria increasing absorption potential of the plant. Nutritional analysis of shoots confirmed the improved iron uptake by siderophore-treated plants. The level of Fe in C138 (BS) and C138 filtrate (S) treatments was even higher than in plants where Fe was provided with synthetic agent (EDDHA) in the full Hoagland solution (Fig. 5). Although the fate of the siderophore has not been evaluated yet, the similar efficiency of the organic and chemical agents appears as a promising alternative to reduce chemical inputs due to Fe chemical fertilization (Orera et al. 2009). A positive side effect was also observed for N, P, K and Na, indicating that a fast recovery of nutrients was occurring in BS-treated plants (Nikolic et al. 2007); this was specially marked for N, consistent with growth parameters, and, therefore, could have an impact in the nutritional quality of edible parts of plants (Briat et al. 2007; Tomasi et al. 2009). Since the siderophore was provided to plants with Fe, effects should only refer to this ion. However, in addition to increased Fe levels on plants treated with the culture filtrate (S) or with the bacteria (BS), a strong side effect is evidenced for aluminium, specially marked in the case of the culture filtrate (S) as compared to the bacterial treatment (BS), and similar to that of full Hoagland solution. This supports the notion of siderophores being able to bind Fe and other metals with variable specificity (Schalk et al. 2011).

It has been described how plants follow two strategies to solubilise unavailable Fe(III) forms from soil. Strategy II refers to the plants which secrete low molecular weight phytosiderophores in order to solubilise and bind iron which is then transported into root cells via membrane proteins (Altomare and Tringovska 2011; Guerinot 2010). In addition, plants manage to select microorganisms able to release siderophores into their rhizosphere (Ramos-Solano et al. 2010), and these bacterial siderophores may be used by both organisms. However, despite its well-demonstrated effect to provide iron to plants (Crowley et al. 1992; Fernández et al. 2005; Johnson et al. 2002), the fate of bacterial siderophores is not clear. According to our results, it seems feasible that the bacterial siderophore is not absorbed by the plant, and iron is obtained through a reduction-based mechanism (Cesco et al. 2002; Hördt et al. 2000; Römheld and Marschner 1986), allowing siderophore release for subsequent re-use. This hypothesis will explain the increase in iron coupled to a higher increase in aluminium in S-treated plants (Fig. 4); since aluminium concentration is not increased in the solution, it means that there is a better efficiency in its absorption on S-treated plants, probably due to a higher affinity for aluminium. Interestingly, the presence of C138 with its siderophores (BS) is more efficient enhancing Fe absorption and preventing potential aluminium toxicity when delivered to the roots, and is consistent with the new role proposed for siderophores on protecting bacteria against metal toxicity (Schalk et al. 2011). However, in order to avoid ion toxicity (aluminium), Fe-bound siderophores could be delivered to the shoot system, since Fe delivery to the plants has been done effectively through the leaves for a long time (Tomasi et al. 2009; Vempati and Loeppert 1988). Furthermore, spraying Fe-free siderophores on leaves could be a way to prevent pathogen attack, based on the Fe-scavenging capacity of bacterial siderophores and the essentiality of Fe for other microorganisms (Duffy and Défago 1999; Schalk et al. 2011) and the reported ability of these molecules to induce systemic resistance (De Vleesschauwer et al. 2008; Meziane et al. 2005). Considering the market potential for Fe chelators (200–400 € ha^–1^ year^–1^ in 2006; Rombolá and Tagliavini 2006), it is worth pursuing this study to evaluate the applicability of the biological iron chelator in field trials.

## Conclusions

Bacterial siderophores from C138 are effective in supplying Fe to iron-starved tomato plants when delivered to the roots, independent of the bacterial presence. Furthermore, results are similar or even better than with full Hoagland solution, representing a promising candidate to develop an organic Fe chelator. The short period needed for fermentation appears as an asset for economic feasibility. In summary, strain C138 tested in this experiment can serve as an effective organic biofertilizer.
